# How do patient demographics, time-related variables, reasons for cancellation, and clinical procedures affect frequency of same-day operating room surgery cancelation? A maximum likelihood method

**DOI:** 10.1186/s12913-018-3247-y

**Published:** 2018-06-15

**Authors:** Omar B. Da’ar, Talal Al-Mutairi

**Affiliations:** 1 Department of Health Systems & Quality Management, College of Public Health & Health Informatics, King Saud Bin Abdulaziz University for Health Sciences, National Guard Health Affairs (NGHA), Riyadh, Saudi Arabia; 2 Graduate School of Professional Studies, St. Mary’s University of Minnesota, Minneapolis, MN USA; 3 King Fahad Hospital, King Abdulaziz Medical City, National Guard Health Affairs (NGHA), Riyadh, Saudi Arabia

**Keywords:** Same-day surgery cancelation, Frequency of cancelation, Probit regression, Saudi Arabia

## Abstract

**Background:**

Cancelation of same-day surgery is a common global problem, wasting valuable hospitals’ operating room (OR) times and imposing significant economic costs. There is limited evidence to support the association between frequency of same-day surgery cancelation and patient demographics, time-related variables, healthcare provider reasons for cancelation, and clinical procedures in Saudi Arabia. The aim of this study was to explore this relationship, providing an understanding of the local context.

**Methods:**

A retrospective cross-sectional study that retrieved medical records to examine the association between the frequency of same-day surgery cancelation and covariates including patient demographics, time-related variables, healthcare provider reason for cancelation, and clinical procedures. The data covered from January 2014 to December 2014 at King Fahad National Guard Hospital in Riyadh. We considered 440 patients that met the inclusion criteria for final analysis. The cancelation was regarded *less frequent* if a patient canceled once in the12 months and *more frequent* if a patient canceled two times or more in the same period. We used descriptive statistics to summarize data and employed a probit regression to estimate the association of frequency of same-day surgery cancelation and covariates via maximum likelihood method. King Abdullah International Medical Research Center granted the institutional approval.

**Results:**

Our study suggests that while reasons of unavailability of OR time were associated with less frequent same-day surgery cancelation, scheduling issues were linked to more frequent cancelations, compared with reasons for patients being unwell on the day of surgery. Waiting time of more than six hours and morning sessions were associated with less frequent cancelations compared to shorter waiting time and afternoon sessions. Compared to general procedures, specialized clinical procedures were associated with cancelations that are more frequent. Further, female patients were more likely to have more cancelations. Finally, being married was associated with the less frequent cancelation of same-day surgery.

**Conclusion:**

Our findings provide evidence of determinants of the frequency of same-day surgery cancelations. This study draws several important implications for hospitals, especially on optimal utilization of resources and minimization of same-day surgery cancellations. The study also offers several recommendations that we believe will spur future research.

## Background

Same-day operating room (OR) surgery cancellation of elective cases is a common global problem in hospitals [1–6] which wastes valuable operating-room time [5]. Many studies have found that reasons for same-day cancellations were mostly related to patients, medical conditions, surgeons, administrative and anesthesia issues [1, 6, 7]. Cancelations of same-day surgery due to these and other reasons frequently occur in hospitals, causing significant economic costs [8] with implications for management in terms of material consumption, medications, and human resources among other factors, as well as causing inconvenience for patients and families [3, 9]. Late cancelations of elective operations have significant psychological, social and financial implications for patients and their families [10]. Hospitals continue to face challenges given that the overall rate of cancellation of elective operations on the day of surgery varies, ranging from 5 to 40% of planned electives [8, 11–16].

Studies in Middle East countries indicate that the issue of same-day cancelation of surgery cases is common [4, 6, 17]. A study in Qatar, for instance, showed late cancelation of up to 15% for outpatient and 13% for inpatient [4]. A study in Saudi Arabia indicated canceled same-day surgery of up to 24% [6], while another in the country showed 8% of surgery cases canceled [17]. Another study in Jordan showed that while Day Unit accounted for 28% of elective operations cancelations, impatient cancelation accounted for 73%, with patient non-attendance and unavailability of hospital beds being the most common reasons [18].

While previous studies have delved into reasons for the day of surgery cancelation, the relationship between the frequency of same-day surgery cancelation, patient demographics, time-related variables, healthcare provider reason for cancelation, and clinical procedures for which a patient had an appointment, have not, at least according to our knowledge, previously been studied in Saudi Arabia. The present study explores this relationship using a combination of statistical methods to estimate the probability of frequency of same-day surgery cancelation. This analysis is noteworthy for two reasons. The present study is the first such analysis of its kind in Saudi Arabia, at least to the best of our knowledge. It is also the first of its kind in that context to employ maximum likelihood estimation with a probit model.

## Methods

A retrospective cross-sectional study that examined medical records of the frequency of cancelation of patients with same-day surgery schedules. The aim was to examine the association between the frequency of same-day surgery cancelation and covariates including patient demographics, time-related variables, healthcare provider reason for cancelation, and clinical procedures for which a patient had an appointment. The retrieved records covered 12 months period from January 2014 to December 2014 at King Fahad National Guard Hospital in Riyadh in the King Abdulaziz Medical City (KFNGH-KAMC).

We considered 440 patients that met the inclusion criteria for final analysis. Information retrieved included time-related variables such as waiting time before cancelation, availability of operating time, and scheduling. Other variables retrieved included reasons for cancelation, clinical procedures, the frequency of cancelation, and patient demographic profiles. The frequency of cancelation was the response variable of interest. We constructed a binary variable where cancelation of same-day surgery was defined as *less frequent* if a patient canceled once in 12 months and *more frequent* if a patient canceled two times or more in the same period. We descriptively summarized all variables of interest and employed a probit regression to estimate the probability of frequency of same-day surgery cancelations with respect to key independent variables using the method of maximum likelihood. The Institutional Review Board (IRB) of King Abdullah International Medical Research Center (KAIMRC) with protocol number-Ref. SP15/148 approved this study.

### Statistical analysis

With a consent, data were retrieved from medical records and then transferred to *STATA Statistical Software Release 12* for analysis [19]. We used descriptive statistics such as means and standard deviations to summarize quantitative variables and frequencies and percentages to characterize categorical variables. We conducted a probit regression analysis by constructing a binary of whether or not patients frequently canceled same-day surgery. We controlled for various factors associated with this probability outcome and descriptively summarized them in Tables 1 and 2.

**Table 1:** Patient demographics and time-related variables (*N* = 440)

Characteristics	Levels	N (%)
Age (Mean ± SD)		46.2 ± 19.7
Waiting time to same-day cancelation in hours (Mean ± SD)		3.6 ± 2.3
Waiting time to same-day cancelation in hours > 6 hours	Waiting time > 6 hours	61 (13.9)
Age	≥18 Years	413 (93.9)
Session of cancelation, n (%)	AM	389 (88.4)
Gender, n (%)	Male	213 (48.4)
Frequency of same-day cancelation in 12 months, n (%)	Once	370 (84.1)
Resident, n (%)	Riyadh	368 (83.6)
Nationality, n (%)	National	392 (89.1)
Marital status	Married	278 (63.3)
	Single	114 (26)
	Divorced	6 (1.4)
	Widowed	18 (4.1)
	Unknown	23 (5.2)
Season		
	Winter	84 (19.1)
	Fasting	19 (4.3)
	Summer	104 (23.6)
	Spring	154 (35.0)
	Fall/Autumn	79 (18.0)

**Table 2:** Patient eligibility, reasons for cancelation, and clinical procedures (*N* = 440)

Characteristics	Levels	N (%)
Eligibility		
	Others	13 (3.0)
	Saudi National Guard (SANG) staff	100 (22.7)
	SANG dependents	230 (52.3)
	KFH employee	15 (3.4)
	KFH employee dependent	14 (3.2)
	Exceptions Saudi	34 (7.7)
	Exceptions 7 diseases	15 (3.4)
	Ineligible/out-of-pocket	19 (4.3)
Reason for cancelation		
	Others	115 (26.1)
	Aspirin intake	14 (3.2)
	No O.R time	56 (12.7)
	Not fasting	13 (3.0)
	Not indicated	51 (11.6)
	Scheduling issues	12 (2.7)
	Uncontrolled blood pressure	37 (8.4)
	Uncontrolled blood sugar	31 (7.1)
	Unwell on day of procedure	79 (18)
	Patient refusal	32 (7.3)
Procedure for same-day surgery		
	Others	234 (53.2)
	Extracapsular cataract extraction & & Intra Ocular Lens (ECCE IOL)	33(7.5)
	Excision Sinus	10 (2.3)
	Hernia repair	49 (11.1)
	Lap Cholecystetomy	30 (6.8)
	Phacoemulsification & Intra Ocular Lens (Phaco IOL)	45 (10.2)
	S/R wisdom teeth	16 (3.6)
	Septoplasty	13 (3.0)
	Tympanoplasty	10 (2.3)

## Results

### Descriptive statistics

Tables 1 and 2 summarize the descriptive statistics of key variables of interest. Among the 440 patients who canceled same-day surgery, 213 (48.4%) were male. The mean age was 42.3 ± 16.7 and supermajority 413 (93.9%) of the patients were 18 years or older. The mean waiting time before cancelation of same-day surgery was 3.6 h (± 2.3 standard deviation). Waiting time before cancelation of same-day surgery was greater than 6 h for 13.9% of the patients. Of the patients who canceled same-day surgery, 389 (88.4%) did so in the morning session. Majority of the patients, 392 (89.1%) were Saudi nationals, 368 (83.6%) live in Riyadh, and 278 (63.3%) were married. Eighty-four percent of patients canceled same-day surgery once in 12 months, while 16% of them canceled more than once in the same period. Nearly a fifth (19.1%) and a fourth (24%) of the patients had canceled same-day surgery during winter and summer respectively. Saudi National Guard (SANG) staff and their dependents constituted nearly three-fourths (76%) of patients canceling same-day surgery. Reasons for cancelation of same-day surgery also included unavailability of OR time (12.7%), scheduling issues (2.7%), uncontrolled blood pressure (8.4%), uncontrolled blood sugar (7.1%), unwell on day of procedure (18%), and patient refusal (7.3%) among other reasons.

### Probit results and analysis

Table 3 shows parameter estimates for the probit model, computed using the method of maximum likelihood estimation. A positive (negative) sign of the coefficients implies that an increase (decrease) in the explanatory variable leads to an increase (decrease) in the predicted probability of frequently canceling same-day surgery elective appointment. It is important to note that our dependent binary variable is reversed-coded (1 = canceled once; 0 = canceled more than once). Therefore, we should interpret negative coefficients or marginal effects as increasing the frequency of same-day surgery cancelation. However, while probit estimation reveals the probabilities and/or the changes in probability, the qualitative nature of such results may present a challenge in interpreting the coefficients. To take into consideration this challenge and give a quantitative interpretation of the probit results, we further present marginal effects as probability units in Table 4.

**Table 3:** Probit Regression: Probability of frequency of cancelation

Probit regression			No. of obs = 357			
			Wald chi2(34) = 42.48			
			Prob > chi2 = 0.1509			
Log pseudo likelihood = − 138.506			R^2^ = 0.1674			
Dependent variable (Frequency of cancelation: 1 = Once, 0 = More than once)	Coefficient	Robust std. Error	Z	P > z	[95% Conf. Interval]	
A						
Waiting time	(Reference is < 6 h)					
Waiting time > 6 h	0.089	0.042	2.1	0.036***	0.006	0.171
Age	(reference = child)					
Adult	0.018	0.437	0.04	0.967	−0.839	0.875
Session	(reference = Afternoon)					
Morning session	0.605	0.278	2.17	0.030***	0.060	1.150
Gender	(reference = Female)					
Male	0.558	0.265	2.11	0.035***	0.039	1.078
Residence	(reference = outside city)					
Riyadh Resident	−0.049	0.255	−0.19	0.849	− 0.548	0.451
Nationality	(reference = non-Saudi)					
Saudi	−1.805	0.566	−3.19	0.001***	−2.915	−0.695
Season	Winter (reference)					
Fasting	1.045	0.599	1.74	0.081	−0.130	2.219
Summer	0.397	0.245	1.62	0.106	−0.084	0.877
Spring	0.642	0.255	2.52	0.012***	0.143	1.142
Fall/Autumn	0.350	0.263	1.33	0.183	−0.165	0.866
Marital status	Married (reference)					
Single	0.012	0.237	0.05	0.96	−0.453	0.477
Divorced	−0.451	0.454	−0.99	0.321	−1.341	0.439
Widowed	−0.139	0.369	−0.38	0.707	−0.863	0.585
Unknown	−1.039	0.427	−2.43	0.015***	−1.877	−0.202
Constant	1.831	0.800	2.29	0.022	0.264	3.399
B						
Eligibility	Saudi dependent (reference)					
Others	−1.258	0.466	−2.7	0.007***	−2.172	−0.344
Saudi	−0.754	0.320	−2.36	0.018***	−1.381	−0.128
Employee	−1.432	0.619	−2.31	0.021***	−2.646	−0.219
Employee dependents	0.221	0.577	0.38	0.701	−0.910	1.353
Exceptions Saudi	−0.511	0.342	−1.49	0.135	− 1.182	0.160
Exceptions 7 diseases	−1.096	0.727	−1.51	0.132	−2.521	0.329
Ineligible/out-of-pocket	−1.301	0.691	−1.88	0.06***	−2.656	0.054
Procedure	Hernia (reference)					
Others	−0.233	0.325	−0.72	0.472	−0.870	0.403
ECCE IOL	−0.019	0.447	−0.04	0.966	−0.895	0.857
Excision Sinus	0.000	(empty)				
Lap Cholecystetomy	−0.571	0.455	−1.26	0.209	−1.463	0.321
Phaco IOL	−0.645	0.379	−1.7	0.089*	−1.388	0.098
S/R wisdom teeth	0.000	(empty)				
Septoplasty	−0.602	0.583	−1.03	0.302	− 1.745	0.541
Tympanoplasty	0.000	(empty)				
Reason for cancelation	Unwell on day of procedure (reference)					
Others	0.046	0.256	0.18	0.857	− 0.456	0.548
Aspirin intake	0.604	0.523	1.15	0.248	−0.422	1.629
No O.R time	1.049	0.443	2.37	0.018***	0.180	1.917
Not fasting	0.000	(empty)				
Not indicated	0.084	0.318	0.27	0.791	−0.538	0.707
Patient refusal	0.125	0.375	0.33	0.739	−0.610	0.859
Scheduling issues	−1.068	0.467	−2.29	0.022***	−1.984	−0.153
Uncontrolled blood pressure	−0.294	0.325	−0.91	0.365	− 0.931	0.343
Uncontrolled blood sugar	0.121	0.324	0.37	0.709	−0.515	0.757
Constant	1.831	0.800	2.29	0.022	0.264	3.399

Significant at ***p < 0.01; **p < .05

Significant at ***p < 0.01; **p < .05; *p < 0.1

**Table 4:** Marginal effects: Patient demographics and Time-related variables

Probit regression			No. of obs = 357			Wald chi2(34) = 42.48	
Log pseudo likelihood = − 138.506			Prob > chi2 = 0.1509			R2 = 0.1674	
Dependent variable = Frequency of cancelation (1 = Once, 0 = More than once)	dF/dx	Robust Std. Err.	Z	P > z	x-bar	[95% C.I.]	
Waiting time	0.019	0.009	2.1	0.036**	3.630	0.002	0.036
Adult	0.004	0.094	0.04	0.967	0.944	−0.180	0.188
Morning session	0.161	0.086	2.17	0.03**	0.880	−0.008	0.330
Male	0.115	0.053	2.11	0.035**	0.459	0.011	0.220
Riyadh Resident	−0.010	0.052	−0.19	0.849	0.832	−0.112	0.091
National	−0.168	0.028	−3.19	0.001**	0.902	−0.223	−0.113
Fasting	−0.293	0.198	−1.74	0.081	0.204	− 0.682	0.095
Summer	−0.163	0.169	−1.1	0.27	0.235	−0.494	0.168
Spring	−0.091	0.143	−0.68	0.497	0.339	−0.371	0.189
Fall/Autumn	−0.183	0.183	−1.17	0.244	0.182	−0.542	0.176
Single	0.002	0.050	0.05	0.96	0.218	−0.095	0.100
Divorced	−0.119	0.142	−0.99	0.321	0.017	−0.398	0.160
Widowed	−0.031	0.089	−0.38	0.707	0.048	−0.206	0.143
Unknown	−0.326	0.165	−2.43	0.015**	0.045	−0.649	−0.003
Surgical procedures (reference = hernia)							
Others	−0.049	0.066	−0.72	0.472	0.557	−0.178	0.081
ECCE IOL	−0.004	0.096	−0.04	0.966	0.092	− 0.192	0.184
Lap Cholecystetomy	−0.153	0.144	−1.26	0.209	0.081	−0.435	0.129
Phaco IOL	−0.174	0.120	−1.7	0.089	0.120	−0.410	0.062
Septoplasty	−0.167	0.198	−1.03	0.302	0.034	−0.555	0.221
Reasons for cancelation (reference = Unwell on day of surgery)							
Others	0.010	0.053	0.18	0.857	0.235	−0.093	0.113
Aspirin intake	0.091	0.051	1.15	0.248	0.039	−0.010	0.191
No O.R time	0.139	0.030	2.37	0.018**	0.126	0.080	0.199
Not indicated	0.017	0.063	0.27	0.791	0.120	−0.105	0.140
Patient refusal	0.025	0.070	0.33	0.739	0.070	−0.112	0.162
Scheduling issues	−0.341	0.181	−2.29	0.022**	0.028	−0.696	0.015
Uncontrolled blood pressure	−0.071	0.088	−0.91	0.365	0.098	−0.242	0.101
Uncontrolled blood sugar	0.024	0.061	0.37	0.709	0.084	−0.095	0.143
Eligibility (reference = Saudi NGHA dependent							
Others	−0.415	0.179	−2.7	0.007***	0.031	−0.765	−0.065
Saudi NGHA	−0.194	0.093	−2.36	0.018**	0.241	−0.377	−0.012
NGHA Employee	−0.483	0.233	−2.31	0.021**	0.028	−0.939	−0.027
NGHA Employee dependents	0.041	0.094	0.38	0.701	0.034	−0.143	0.226
Exceptions Saudi	−0.134	0.105	−1.49	0.135	0.078	−0.341	0.072
Exceptions 7 diseases	−0.350	0.283	−1.51	0.132	0.036	−0.905	0.205
Ineligible/out-of-pocket	−0.430	0.266	−1.88	0.06**	0.036	−0.951	0.091

Obs. *P* = 0.823529

Pred. *P* = 0.870344 (at x-bar)

Canceled once = 1; canceled more than once = 0; Significant at ***p < 0.01; **p < .05

(*) dF/dX is for discrete change of dummy variable from 0 to 1; z and p > |z| are the test of the underlying coefficient being 0

Canceled once = 1; canceled more than once = 0

### Marginal effects

In order to give a quantitative interpretation of the probit results, we present average or marginal effects as probability units (Table 4). The probability of frequency of same-day surgery cancelation in a morning session was 0.16 less than an afternoon (*P* = 0.03 < 0.05). The probability of frequency of same-day surgery cancelation by a male patient was 0.12 less than a female patient, while the probability of frequent cancelation of a same-day surgery by a Saudi patient was 0.17 more than a non-Saudi (*P* = 0.035 < 0.05; (*P* = 0.001 < 0.05 respectively). The probability of same-day surgery cancelation during the month of fasting was 0.29 more than during winter season. This result was however not statistically significant. The probability of frequency of same-day surgery cancelation if patients waited for more than 6 h was 0.02 less than if patients waited for less than 6 h before surgery (*P* = 0.036 < 0.05). The probability of frequency of same-day surgery cancelation of a patient whose marital status is unknown, or not reported was 0.33 more compared to married patients (*P* = 0.015 < 0.05). Table 4 depicts the summary of these results.

As presented in Table 4, the probability of same-day surgery cancelation for a patient scheduled for Phaco IOL procedure is 0.17 more than a patient scheduled for Hernia. While this result is not statistically significant, there is evidence that procedures such as Phaco IOL are more frequently canceled, especially in the ophthalmic literature due to reasons for a patient not being fit for local anesthesia or pre-existing conditions [20]. The probability of same-day surgery canceled more frequently for OR time availability is 0.14 less than if the reason was patient being unwell on the day of the procedure (*P* = 0.018 < 0.05). However, the probability of same-day surgery canceled more frequently for scheduling issues is 0.34 more than if the reason was patient being unwell on the day of the procedure (*P* = 0.022 < 0.05). The probability of same-day surgery canceled more frequently is higher among SANG patients, hospital employees, and patients who are ineligible or pay out-of-pocket compared to dependents of SANG members.

## Discussion

In this study, we attempted to examine the association between the probability of frequency of same-day surgery cancelation and various covariates such as patient demographics, time-related variables, healthcare provider reasons for cancelation, and clinical procedures for which patients had appointments. This relationship has not hitherto been studied either prospectively or retrospectively in Saudi Arabia. We employed maximum likelihood estimation with a probit model to estimate that relationship. Probit model estimations using a maximum likelihood procedure have been widely used in healthcare studies, but few examples will suffice. Probit estimation has previously been used in assessing factors associated with quality of services and demand for health care [21], demand for specialty drugs [22], and association of weight and height with a timing of deciduous tooth emergence [23]. Probit approach has also been used to examine awareness and acceptability of human papillomavirus vaccine [24], and prevalence of total diagnosed and undiagnosed diabetes [25].

Previous studies have shown that the issue of same-day cancelation of surgery cases is common [4, 6, 17]. Previous studies also have shown that cancellations of short-stay cases in hospitals frequently occur, causing burden and challenges for theater and surgical operating time [26–28].

Consistent with other studies [29], our study demonstrated that unavailability of OR time is a common cause of cancelation of same-day surgery. However, our study specifically showed that unavailability of OR time is less likely to be associated with the frequent cancelation of same-day surgery relative to a patient being unwell on the day of surgery. This may imply that frequency of cancelation has to do more with patient-related reasons than the capacity of the facility. Additionally, our study revealed more frequent same-day surgery cancelations for scheduling issues compared with reasons for being unwell on the day of the procedure. Findings of previous studies have attributed scheduling issues to day of surgery cancelation [3, 16, 30]. Moreover, the present study showed that morning session and waiting time of more than 6 h were associated with less frequent cancelation. The finding with respect to sessions is somewhat intuitive and relevant to local customs and climatic conditions such as high temperatures, which may affect working shifts. There are typically fewer activities during afternoon sessions in the region due to very high temperatures. Although more waiting time inconveniences patients, it appears also to give them hope that the facility may honor their appointments for same-day surgery, hence less frequent cancelation.

Like previous studies, our study found that demographic factors play an important role in determining the frequency of same-day surgery cancelation. For instance, our study suggested that female cancel same-day surgery more frequently than male. Cultural issues in Middle Eastern countries such as male family members having the latitude to decide on the movement of women may explain this result. Limitations on the physical mobility of women and girls provide hindrances for them to successfully access and use health services and affect their health-seeking behaviors [31]. That said, the conclusion of previous studies on gender difference with regard to frequency of cancelation is mixed. While some studies showed a higher proportion of female cancelations [16], others revealed no differences between the frequency of cancelation of males and females [32].

This study indicates that married patients less frequently canceled same-day surgery cases compared with patients whose marital status was unknown or not revealed. This finding may be intuitive and consistent with local cultural practices. For instance, the movement of unmarried or unaccompanied women is limited. A close male relative is crucial for their company including an elective surgery.

Insofar as clinical conditions are concerned, our study illustrated that specialized procedures have higher probabilities of same-day surgery cancelation compared to procedures that are more general. This finding is somewhat in agreement with studies that suggested that because the odds of unavailability of operating time were significantly less in general surgery, there were fewer cancelations compared with more specialized procedures such as orthopedics, otolaryngology, neurosurgery, pediatrics, gynecology, ophthalmology, and dentistry [33]. In addition, our finding is consistent with studies confirming evidence of some specialties being more susceptible to the day of surgery cancelations [34].

Our study potentially adds to the growing literature on same-day surgery in two fundamental ways. The present study is the first such analysis of its kind in Saudi Arabia that employed maximum likelihood estimation with probit model to examine factors associated with the probability of same-day surgery cancelation. Similarly, our study contributes to the understanding of the crucial role of context and local application, especially in the wake of the dearth of studies dealing with the frequency of same-day surgery cancelation in the region. In this context, our study provides workable evidence that we believe is an important strategy for optimizing resource usage and management of same-day surgery cancelations by hospitals in the country and across the region. Resource management and its implication on cost, as pointed out in a recent review, is an area that needs sensitization in Saudi Arabia [35].

### Limitations

It is important, however, to note that our study has some limitations. For instance, there may be biases due to errors of entry, or misclassification, given that the data utilized for the present study is from retrospective medical records. As is common with retrospective studies, we cannot ascertain the existence (or lack thereof) of confounding factors in the data. There are various methods of dealing with such problems, including randomization. We based our study on the availability of retrospective data, but such methods are typically applied at the time of study design. Our study did not directly deal with patients, rendering randomization untenable at the start. Additionally, we should note that while our study found an association between the probability of frequency of same-day surgery cancelation and the various factors relating to patient demographics and clinical conditions, we could not establish causality given that the retrospective data were cross-sectional. Moreover, our study was limited to the OR of a single hospital. Perhaps inclusion or comparison with other facilities may exhibit different distributions and associations between variables of interest.

## Conclusion

This study explored the association between the probability of frequency of same-day surgery cancelation and various factors such as patient demographics, time-related variables, healthcare provider reasons for cancelation, and clinical procedures. The study employed a combination of descriptive statistics and maximum likelihood estimation with probit model for inferential analysis. The study found a significant association between the probability of the frequency of same-day surgery cancelations and some key covariates of interest. For example, the present study revealed that facility characteristics-related issues such as availability of operating room time and scheduling do associate with whether or not patients canceled same-day surgery cases. In particular, while reasons of unavailability of OR times were associated with the less frequent cancelation of same-day surgery, scheduling issues were linked to cancelations that are more frequent. In addition, we found that waiting time of more than 6 h and morning sessions were associated with less frequent cancelation compared to less waiting time and afternoon sessions. Moreover, the study showed evidence of some highly specialized clinical procedures being more susceptible to the day of surgery cancellations compared to general procedures. Further, the study indicated significant gender difference in the frequency of same-day surgery cancelations, with female patients being more likely to cancel same-day surgery appointments.

We draw several implications that we believe are important for hospital operating rooms management, especially on optimal utilization of resources and minimization of same-day surgery cancelations. First, with regard to issues such as scheduling, waiting time, and sessions of operation, hospital facilities need to incorporate patient preferences, reduce waiting time by regularly reviewing their staffing policies, workflows, and shift system. Hospitals should occasionally form task forces to understand, investigate, and establish whether indeed the factors associated with frequency of same-day surgery cancelations are facility-specific, the result of human errors, or even system-wide phenomena. For instance, since scheduling normally should focus on patients rather than providers, understanding these issues further will help facilities institute measures that ensure better care. Moreover, hospitals need to find ways of accommodating or rerouting patients requiring more specialized same-day surgery in order to minimize potential complications, or even death arising from the cancelation of such procedures.

We offer several recommendations that we believe will spur future research. First, there is a need for further research that isolates facility or OR-specific practice style as natural experiments in order to assess the comparative effectiveness of different operating rooms across hospitals in the locality. In addition, although we found other literature in the region linking gender differences in frequency of cancelation to cultural reasons, there is a need for further studies to establish the extent to which such attribution affects demand for health care in the facility or country. Furthermore, in order to address the limitation of retrospective cross-section data, possibly establish causality of the covariates on the frequency of same-day cancelation, we recommend a robust longitudinal study undertaking that follows patients’ records over multiple periods. Additionally, in order to deal with retrospective data-related biases and the possibility of the presence of confounding factors, we recommend future studies to consider randomization or other methods of minimizing such challenges when designing studies.

## Abbreviations

DOS: Day of surgery
ECCE: Extracapsular cataract extraction
IOL: Intra Ocular Lens
IRB: Institutional Review Board (IRB)
KAIMRC: King Abdullah International Medical Research Center
KFNGH: King Fahad National Guard Hospital in Riyadh
KMAC: King Abdulaziz Medical City
OR: Operating room
Phaco: Phacoemulsification
SANG: Saudi National Guard
